# OPTIMAL MEDICATION MANAGEMENT IN ALZHEIMER’S DISEASE AND DEMENTIA

**DOI:** 10.1093/geroni/igz038.046

**Published:** 2019-11-08

**Authors:** Ariel Green

**Affiliations:** Johns Hopkins University, Baltimore, Maryland, United States

## Abstract

For older individuals with Alzheimer’s disease and related dementias (ADRD) and multiple chronic conditions (MCC), taking more medications is associated with greater risk of adverse drug events, drug interactions, treatment burden, and cognitive changes from medication side effects. Optimizing medication through deprescribing (the process of reducing or stopping the use of inappropriate medications or medications unlikely to be beneficial) can help avoid adverse drug effects and improve outcomes for MCC patients, particularly for those with ADRD. Findings to date are limited to primarily Caucasian patients. This talk will focus on work geared to elicit perspectives on medication use, communication about medication, and deprescribing among African American and Hispanic older adults with ADRD and MCC, their family members, and clinicians caring for these populations.

